# Induction of Durable Antitumor Response by a Novel Oncolytic Herpesvirus Expressing Multiple Immunomodulatory Transgenes

**DOI:** 10.3390/biomedicines8110484

**Published:** 2020-11-09

**Authors:** Dmitry V. Chouljenko, Jun Ding, I-Fang Lee, Yanal M. Murad, Xuexian Bu, Guoyu Liu, Zahid Delwar, Yi Sun, Sheng Yu, Ismael Samudio, Ronghua Zhao, William Wei-Guo Jia

**Affiliations:** Virogin Biotech Canada Ltd., 408-3800 Wesbrook Mall, Vancouver, BC V6S 2L9, Canada; jding@virogin.com (J.D.); elee@virogin.com (I.-F.L.); ymurad@virogin.com (Y.M.M.); xxbu@virogin.com (X.B.); gyliu@virogin.com (G.L.); zdelwar@virogin.com (Z.D.); jsun@virogin.com (Y.S.); syu@virogin.com (S.Y.); isamudio@virogin.com (I.S.); rzhao@virogin.com (R.Z.); wjia@virogin.com (W.W.-G.J.)

**Keywords:** immunotherapy, oncolytic virus, herpes simplex virus, cancer vaccine, antitumor immunity, combinatorial therapy, immune checkpoint blockade, interleukin-12, interleukin-15, VG161

## Abstract

Oncolytic virotherapy is a promising new tool for cancer treatment, but direct lytic destruction of tumor cells is not sufficient and must be accompanied by strong immune activation to elicit anti-tumor immunity. We report here the creation of a novel replication-competent recombinant oncolytic herpes simplex virus type 1 (VG161) that carries genes coding for IL-12, IL-15, and IL-15 receptor alpha subunit, along with a peptide fusion protein capable of disrupting PD-1/PD-L1 interactions. The VG161 virus replicates efficiently and exhibits robust cytotoxicity in multiple tumor cell lines. Moreover, the encoded cytokines and the PD-L1 blocking peptide work cooperatively to boost immune cell function. In vivo testing in syngeneic CT26 and A20 tumor models reveals superior efficacy when compared to a backbone virus that does not express exogenous genes. Intratumoral injection of VG161 induces abscopal responses in non-injected distal tumors and grants resistance to tumor re-challenge. The robust anti-tumor effect of VG161 is associated with T cell and NK cell tumor infiltration, expression of Th1 associated genes in the injection site, and increased frequency of splenic tumor-specific T cells. VG161 also displayed a superb safety profile in GLP acute and repeated injection toxicity studies performed using cynomolgus monkeys. Overall, we demonstrate that VG161 can induce robust oncolysis and stimulate a robust anti-tumor immune response without sacrificing safety.

## 1. Introduction

A diverse range of oncolytic viruses (OVs) has shown efficacy in preclinical studies (reviewed in [1,2]). However, only two OVs have cleared the hurdle of regulatory approval, consisting of the adenovirus H101 approved in China to treat head and neck cancer (4) and Talimogene laherparepvec (T-VEC) approved by the FDA for the treatment of advanced melanoma. It has become clear that direct infection and lysis of tumor cells is often not sufficient to generate a durable anti-tumor response. T-VEC was optimized for immunotherapy by expressing the cytokine granulocyte macrophage colony-stimulating factor (GM-CSF) and can generate systemic anti-tumor immunity as evidenced by observations of tumor regression in noninjected lesions [3,4,5,6,7]. However, the overall durable response rate in patients treated with T-VEC is below 20% [5,7]. Therefore, it is apparent that a more robust immune response must be elicited in addition to potent oncolysis for an OV therapy to achieve long-lasing efficacy in the clinic.

In the present study, we have constructed a novel oncolytic herpes simplex virus (VG161) capable of delivering four immunomodulatory molecules into the tumor microenvironment, consisting of IL-12, IL-15, IL-15 receptor alpha subunit isoform 1 (IL-15RA), and a fusion protein (TF-Fc) capable of blocking PD-1/PD-L1 interactions [8]. IL-12, IL-15, and immune checkpoint inhibitors have individually shown great potential as anti-cancer therapeutics, and antibody-based checkpoint inhibitors are readily available and display enhanced anti-tumor activity when co-administered with T-VEC or with oncolytic Newcastle disease virus [9,10]. IL-12 has a potent ability to activate T and NK cells, along with many other immunomodulatory effects, and it has demonstrated significant therapeutic efficacy in many tumors [11,12], especially when administered intratumorally [13,14]. Notably, production of IL-12 by tumor-infiltrating dendritic cells was shown to sensitize tumors to anti-PD-1 treatment and stimulate antitumor T cell immunity [15]. While IL-15 has also shown promise as a monotherapy in a variety of tumor models by facilitating induction of CD8+ T cells and NK cells, its antitumor effect is synergistically enhanced when administered in combination with other cytokines, including IL-12 [16,17]. Importantly, we have previously shown that IL-15 synergizes with components of the HSV-1 molecular structure to induce potent activation of NK cells, leading to increased cytolysis of cancer targets [18,19]. VG161 was also engineered to express IL-15RA to replicate the natural trans-presentation of IL-15 and to increase the potency of IL-15 signaling due to prolonged half-life of IL-15 when complexed with IL-15RA (reviewed in [20]). Inclusion of the PD-L1 blocker was designed to counteract the inhibitory effect of PD-1/PD-L1 interaction on CD8+ T cells and NK cells, leading to greatly enhanced anti-tumor immunity (reviewed in [21]). VG161 additionally carries a deletion in the viral gene encoding ICP34.5 as a safety measure to abrogate neurovirulence and an intact ICP47 protein to enhance virus persistence and extend the window of time for payload delivery.

In vitro testing revealed a cooperative immunostimulatory effect between IL-12, IL-15, and the PD-L1 blocker. Intratumoral injection of VG161 induced significant tumor regression and prolonged survival in both syngeneic and human xenograft mouse models. Treatment with VG161 elicited robust anti-tumor immunity, acting in concert with the anti-viral and adjuvant effects of OV treatment to elicit a durable anti-tumor immune response far superior to that exerted by the backbone virus VG160. VG161 promoted infiltration of T and NK cells, expression of Th1 genes, increased the frequency of tumor-specific T cells in the spleen, induced abscopal responses in syngeneic tumor models and provided resistance to tumor re-challenge. Moreover, toxicity studies performed in monkeys validated the safety of VG161 when given in either single or repeated doses. Our results demonstrate that by combining potent oncolytic activity with a broad spectrum of immune-stimulatory payloads, VG161 can promote long-lasting anti-tumor immunity.

## 2. Materials and Methods

### 2.1. Cell Lines

African green monkey kidney (Vero) cells, the human tumor cell lines H460, U87, MCF-7, LS174T, and MDA-MB-231, and the mouse tumor cell lines 4T1, B16-F10, CT26, and A20 were obtained from the American Type Culture Collection (Manassas, VA, USA). Cells were maintained in Dulbecco’s modified Eagle’s medium (DMEM) (Gibco-BRL, Grand Island, NY, USA) supplemented with 10% fetal bovine serum (FBS) (Thermo Fisher Scientific, Waltham, MA, USA).

### 2.2. Construction and Characterization of PD-L1 Blocker Peptide

A peptide array method was used to discover and chemically synthesize the human variant of the TF peptide from fragments of human PD-1 protein. Peptide array results were generated by incubating derivative test peptide-containing membranes with 15 μg/mL of negative control human IgG or recombinant human PD-L1 Fc at 4 °C, overnight. The resulting signals were detected by HRP-conjugated anti-human IgG and HRP substrate. The mouse version of this peptide was designed based on the human TF peptide, with several amino acids replaced to achieve a closer match to the mouse PD-1 sequence. Mouse TF peptide candidates were screened by ELISA to find one that binds to mouse PD-L1 more effectively than the human TF peptide. Human TF peptide (TAHPSPSPRSAGQF) was fused to human IgG4 Fc (TF-Fc), while the mouse TF peptide (TRYPSPSPKPEGRF) was fused to mouse IgG1 Fc to enhance peptide stability and to facilitate detection of the fusion protein.

### 2.3. Recombinant Virus Construction

**Figure 1:**
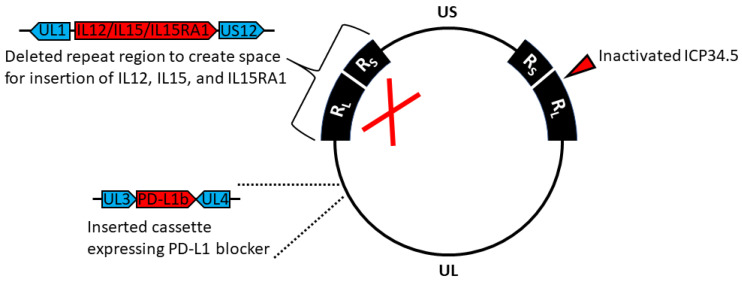
**Genomic map of VG161.** Prototypic arrangement of the wild-type HSV-1 genome with the unique long (UL) and unique short (US) regions flanked by inverted repeats R_L_ and R_S_, respectively. Expanded regions indicate modifications made to the HSV-1 genome during construction of VG161.

Herpes simplex virus type 1 (HSV-1) strain 17 was used as the backbone for construction of all viruses. All viral mutagenesis with the exception of ICP34.5 deletion was performed in *Escherichia coli* using standard lambda Red-mediated recombineering techniques implemented on the HSV-1 strain 17 genome cloned into a bacterial artificial chromosome (BAC). The HSV-345 virus backbone lacking both copies of the gene encoding ICP34.5 was constructed by recombination in transfected mammalian cells. An expression cassette for the secretable PD-L1 blocking peptide TF conjugated to human IgG4 Fc (TF-Fc) controlled by the human EF-1α promoter was inserted between viral genes UL3 and UL4, and the terminal repeat region was completely replaced by an expression cassette for human IL-12, IL-15, and IL-15 receptor alpha subunit isoform 1 (IL-15RA) driven by the CMV promoter and with each element separated by self-cleaving P2A peptides to create hVG161 (VG161) (Figure 1).

Additional HSV-1 mutants were constructed for testing purposes, including a variant (VG160) which does not express IL-12, IL-15, IL-15RA, or the PD-L1 blocker peptide but contains all other modifications present in VG161. To facilitate in vivo testing in a variety of mouse models, we constructed mVG161, which is identical to VG161 apart from mouse IL-12 replacing human IL-12 and the presence of a mouse-specific version of the PD-L1 blocker peptide conjugated with mouse IgG1 Fc. Human IL-15 was retained in mVG161 due to its cross-reactivity to mouse cells [22]. The VG-VEC mutant was constructed by inserting an expression cassette for human granulocyte-macrophage colony stimulating factor (GM-CSF, GenBank accession M11220) into the deleted terminal repeat region of VG160.

The resulting mutant BACs were isolated using the Qiagen HiSpeed MidiPrep Kit (Qiagen, Frederick, MD, USA) and transfected into Vero cells to recover the virus using Lipofectamine 2000. Targeted sequencing of all modified regions and restriction profiling was used to verify genomic integrity.

### 2.4. Cytotoxicity Assay

Tumor cells were infected with the indicated viruses at MOI of 0.04, 0.2, and 1 for 72 h, and cytotoxicity was assessed using MTT (3-[4,5-dimethylthiazol-2-yl]-2,5 diphenyl tetrazolium bromide) assay performed using standard protocols as described in [23].

### 2.5. Replication Kinetics of Mutant Viruses

Nearly confluent H460 and MCF-7 human cancer cell monolayers grown in 12-well plates were infected with the indicated viruses at MOI = 0.1, using a separate plate for each timepoint. Infection was carried out in serum-free DMEM while shaking the plate at 20 rpm in 4 °C for 1 h, then transferring to 37 °C and incubating for 1 h to allow the virus to penetrate, followed by removal of the virus-medium mixture and washing 3 times with PBS and once more with serum-free DMEM to remove residual extracellular virus. Infected cell monolayers were overlaid with DMEM supplemented with 2% FBS and incubated until the indicated timepoints at 37 °C and 5% CO^2^. The entire contents of each well (including cells and supernatant) were harvested at 0, 12, 24, 36, and 48 h by freezing the entire plate at −80 °C. Harvested samples were subjected to 3 freeze-thaw cycles to release cell-associated virus, followed by centrifugation to remove cell debris. The supernatant was stored at -80°C for titration by plaque assay.

Plaque assay was carried out in nearly confluent Vero cell monolayers grown in 12-well plates. Briefly, each well of a 12-well plate was seeded with 3 × 10^5^ Vero cells in 1 mL of DMEM supplemented with 10% FBS and incubated overnight at 37 °C and 5% CO_2_ until the cells reached ~95% confluency, followed by infection for 1 h with serially diluted virus in serum-free DMEM. The virus inoculum was subsequently aspirated and 1.5 mL of DMEM containing 1% methylcellulose was added to each well. Plates were incubated at 37 °C and 5% CO_2_ until plaques were visible. Infected cells were fixed with 4% glutaraldehyde and stained with 2% crystal violet (hexamethyl pararosaniline chloride). Plaques were counted using an inverted light microscope at low magnification.

### 2.6. Enzyme-Linked Immunosorbent Assay (ELISA)

The amount of human IL-12p70, human IL-15/IL-15RA complex, or PD-L1 blocker produced from VG161-infected cell supernatant was quantified using the following ELISA kits: Human IL-12p70 ELISA MAX Deluxe (BioLegend, San Diego, CA, USA), IL-15 Human Uncoated ELISA Kit (Thermo Fisher Scientific, Waltham, MA, USA), and Human IgG4 ELISA kit (Thermo Fisher Scientific, Waltham, MA, USA). Two samples per virus per cell type were analyzed, and supernatants from VG160-infected tumor cells were used as negative controls, while the recombinant standard protein from the ELISA kit that was used to generate the standard curve served as the positive control. To detect human IL-2 secretion, human IL-2 ELISA kit was used (Thermo Fisher Scientific, Waltham, MA, USA). Briefly, target recognizing antibody was coated in 96-well Immuno Maxisorp flat-bottom plate. The binding of target was detected via a biotinylated monoclonal antibody, streptavidin-horseradish peroxidase (HRP), and 3,3′,5,5′-Tetramethylbenzidine (TMB) substrate. Color development was stopped by adding 1 M H_2_SO_4_. Absorbance measurements were collected at 450 and 570 nm wavelengths via microplate reader (Molecular Devices, San Jose, CA, USA).

The amount of mouse IL-12p70 and human IL-15/IL-15RA complex produced from four samples of mVG161-infected Vero cell supernatant was quantified using the following ELISA kits: Mouse IL-12p70 ELISA MAX Deluxe (BioLegend, San Diego, CA, USA) and Human IL-15/IL-15 R alpha Complex DuoSet ELISA (R&D Systems). Supernatant from uninfected Vero cells was used as the negative control, while the recombinant standard protein in the ELISA kit used to generate the standard curve served as the positive control. Briefly, target recognizing antibody was coated in 96-well Immuno Maxisorp flat-bottom plate. The binding of target was detected via a biotinylated monoclonal antibody, streptavidin-horseradish peroxidase (HRP), and 3,3′,5,5′-Tetramethylbenzidine (TMB) substrate. Absorbance measurements were collected at wavelength of 450 nm via a microplate reader (Molecular Devices, San Jose, CA, USA). For mouse PD-L1 blocker expression, anti-mouse IgG antibody (Sigma, St. Louis, MO, USA) was coated on the wells of ELISA plates at 4 °C overnight. On the following day, virus-infected supernatant was applied to ELISA wells and the binding mouse PD-L1 blocker was detected by an HRP-conjugated, anti-mouse IgG antibody (PerkinElmer, Waltham, MA, USA) and TMB substrate.

Supernatants harvested from cell-based assay were analyzed for IFN-γ production by ELISA using matched Ab pairs for IFN-γ (Thermo Fisher Scientific, Waltham, MA, USA) according to the manufacturer’s recommended procedure.

### 2.7. IL-2 Assay

293FT cells were transfected with TF-Fc-containing vector for 48 h and supernatant was harvested for the following assay. 5 × 10^4^ Jurkat T cells were activated with 1 µ/mL of PHA and 50 ng/mL of PMA and co-cultured with 1 × 10^5^ PD-L1-expressing tumor cells mixed with PD-L1 blocking peptide-containing supernatants at 37 °C for 48 h. After 48 h, cell culture supernatants were harvested and IL-2 production from Jurkat T cells was assessed by IL-2 ELISA (Thermo Fisher Scientific, Waltham, MA, USA).

### 2.8. In Vitro Quantification of the Combinatorial Effect between IL-12, IL-15/IL-15RA and PD-L1 Blocker

A total of 1.5 × 10^7^ human PBMCs were pre-activated with 2.5 ug/mL of Phytohemagglutinin (PHA) in a 37 °C incubator for 48 h. PHA-activated cells were subsequently seeded at density of 2 × 10^4^ cells/well in a 96-well, U-bottom plate in the presence of VG161-infected cell supernatant and 0.4 µg of PD-L1 recombinant protein. To examine the effect of IL-12 and IL-15/IL-15RA complex, 0.5 µg of neutralizing anti-IL-12 antibody or 0.5 µg of anti-IL-15 antibody were added into the co-incubation mixture. To examine the effect of PD-L1 blocker, VG161-infected supernatant was mixed with Dynal beads in a 1.5 mL microtube and incubated at 4 °C for 60 min with rotation. After incubation, the microtube was placed in magnet and unbound supernatant was used to co-incubate with PHA-activated cells. Supernatants were harvested 48 h after co-incubation and human IFN-γ production was assessed by ELISA assay.

### 2.9. In Vivo Tumor Models

All experimental animal procedures were approved by the BRI Biopharmaceutical Research Inc. Animal Care Committee and followed the guidelines and policies of the Canadian Council on Animal Care (protocol number AUP-2016-001 approved on 15 April 2016). Tumor cells were harvested while in exponential growth phase (approximately 80–90% confluence) using 0.25% trypsin (Thermo Fisher Scientific, Waltham, MA, USA). Cells were suspended in DMEM supplemented with 10% FBS prior to cell counting, then centrifuged at 225× *g* at 4 °C for 10 min. Cell pellets were resuspended in PBS with 60% matrix gel (Corning Inc., Corning, NY, USA) to a concentration of 2 million cells/100 µl and stored on ice. Tumor cells were subcutaneously implanted into the lower right flank of each mouse using 1 × 10^6^ CT26 cells/mouse, 2 × 10^6^ LS174T cells/mouse, and 5 × 10^6^ A20 cells/mouse flank. Observation of tumor growth was conducted every day after implantation and tumor volume was measured using a digital caliper 3 times/week and calculated using the following formula: Tumor Volume = 1/2 × a × b2 (a = longest diameter in mm, b = shortest diameter in mm). The virus was inoculated into the tumor tissue when tumor size exceeded 100 mm^3^.

### 2.10. Flow Cytometric Analysis

Tumors were harvested from mice and single cell suspensions were made by incubating minced tumor at room temperature for 1 h in enzyme digestion buffer containing type V collagenase, type IV DNase, and type V Hyaluronidase. Cells were washed and incubated for 30 min on ice in staining buffer (Thermo Fisher Scientific, Waltham, MA, USA) with different combinations of the following antibodies: FITC-conjugated anti-CD4 (RM4-5), anti-CD8a (53-6.7), anti-Gr-1 (RB6-8C5), PE-conjugated anti-49b (DX5), anti-CD8a (53-6.7), PerCP-Cy5.5-conjugated anti-CD45 (30-F11), APC-conjugated anti-CD3 (17A2), and anti-CD11b (M1/70) antibodies (Thermo Fisher Scientific, Waltham, MA, USA). For intracellular staining of FoxP3, cells pre-stained with antibodies targeting surface proteins were incubated with 1× fixation/permeabilization buffer and subsequently stained with PE-conjugated anti-FxoP3 (FJK-16s) antibody in 1× permeabilization buffer (Thermo Fisher Scientific, Waltham, MA, USA). FACS analysis was performed on a NovoCyte 2000 Flow Cytometer using NovoExpress software (ACEA Biosciences, San Diego, CA, USA).

### 2.11. Detection of Tumor Infiltrating Lymphocytes

Mouse tumor sections were glass mounted and deparaffinized. For double immunofluorescence staining, sections were incubated in 3% skim milk in PBS-T for 30 min and then incubated overnight at room temperature with a combination of two primary antibodies. These combinations were rat monoclonal anti-CD3 antibody (Abcam 1:100)/rabbit polyclonal anti-HSV antibody (DAKO 1:100), and rat monoclonal anti-perforin antibody (Abcam 1:100)/rabbit polyclonal anti-HSV antibody (DAKO 1:100). Sections were then incubated with a mixture of fluorophore-labeled secondary antibodies (Alexa Fluor 488 goat anti-RAT and Alexa Fluor 546 goat anti-rabbit, Invitrogen, Burlington, Ontario, Canada; 1:500), counterstained with Hoechst (Sigma, St Louis, MO, USA), and mounted on glass slides prior to imaging.

### 2.12. ELISpot T Cell Activity Assay

Mouse IFN-γ ELISPOT assays (Mabtech Inc., Cincinnati, OH, USA) were performed according to the manufacturer’s instructions. Briefly, splenocytes isolated from treated mice were added to each well (100,000 cells/well) and stimulated overnight with CT26 cells (5000 cells/well) to detect CT26-specific responses. Results were expressed as the number of spots per well.

### 2.13. Gene Expression Analysis

Gene expression analysis was performed with real-time RT-PCR. A total of 5 × 10^7^ PFU of VG161 was intratumorally administered to mice bearing the CT26 tumors. Tumors were collected 24 h after the virus injection, and the mRNA was isolated using RNeasy Plus isolation kit (Qiagen, Frederick, MD, USA). RT-PCR amplification was performed using the RT^2^ First Strand Kit. We used the RT^2^ Profiler™ PCR Array Mouse Innate & Adaptive Immune Responses (PAMM-052Z) with 84 mouse genes involved in the host innate and adaptive immune response for the gene expression profiling, and RT^2^ SYBRR Green qPCR Master Mix (all from Qiagen, Frederick, MD, USA) was used for setting up the qPCR reactions. Thermal cycling was performed using ABI-7000 (Applied Biosystems, Foster, CA, USA). Data analysis was performed, and a heat map was generated using online analysis tools available on the Qiagen data analysis center.

### 2.14. Biodistribution Analysis

Biodistribution analysis of VG161 was performed on nude mice implanted with LS174T cells. Briefly, tumors were injected with 5 × 10^7^ pfu of VG161, either once (single injection) or 3 times (3 injections on 3 consecutive days) and different organs were collected at the indicated time points. DNA was isolated using the DNeasy Blood and Tissue Kit (Qiagen, Frederick, MD, USA) and viral copies were measured by qPCR using primers and probe specific to the codon optimized IL-15RA1 gene which is unique to VG161. Specificity of the qPCR assay was validated, and no cross-reactivity was observed with either human or mouse genomic DNA. The viral copy numbers were calculated per µg of genomic DNA.

### 2.15. Primate Toxicity Studies

All primate toxicity studies were conducted in JOINN Laboratories (Beijing) in compliance with the current United States Food and Drug Administration (FDA) Good Laboratory Practice (GLP) Regulations, 21 Code of Federal Regulations (CFR) Part 58, and China Food and Drug Administration (CFDA) GLP regulations (CFDA Executive Order 34, September 2017). Animal care was compliant with the relevant Standard Operating Procedures (SOPs) of JOINN Laboratories, the Guide for the Care and Use of Laboratory Animals, 8th Edition (Institute of Laboratory Animal Resources, Commission on Life Sciences, National Research Council; National Academy Press; Washington, DC, USA, 2010), and the U.S. Department of Agriculture (USDA) through the Animal Welfare Act (Public Law 99–198). JOINN Laboratories is fully accredited by the Association for Assessment and Accreditation of Laboratory Animal Care International (AAALAC). Procedures used in this study were approved by the Institutional Animal Care and Use Committee (IACUC) at JOINN Laboratories. The single dose toxicity study (IACUC serial number ACU18-403, study number P18-021-JD) was initiated on 16 April 2018 and the 4-week toxicity study (IACUC serial number ACU18-950, study number P18-021-CD) was initiated on 8 August 2018 under animal use license number SYXK (Jing) 2016-0029 (Expiration Date: 8 August 2016 to 8 August 2021) authorized by the Beijing Science & Technology Commission.

GLP acute (single dose) and repeated injection toxicity studies were performed using cynomolgus monkeys (*Macaca fascicularis*). A total of 8 monkeys (4 animals/sex) were used for the acute toxicity study and were assigned to 4 groups (1 animals/sex/group) based on body weights in both sexes. The monkeys were treated via single intramuscular injection (according to standard GLP toxicity study protocol for local injection) with vehicle control or VG161 at doses of 2.97 × 10^7^, 2.97 × 10^8^, and 2.97 × 10^9^ PFU/animal, respectively, followed by a 14-day observation period. Parameters evaluated in the study included mortality/morbidity, clinical signs, body weights, body temperature, electrocardiogram, clinical pathology (hematology, coagulation, clinical chemistry, and urinalysis), T-lymphocyte, cytokines, vector absorption, expression products in serum, tissue distribution of viral DNA, and macroscopic examinations. All the animals were euthanized on Day 15 and received a complete necropsy examination.

A 4-week toxicity study was also performed by administering repeated intramuscular injections to cynomolgus monkeys. The monkeys (*n* = 7 animals/sex/group) were treated via repeated intramuscular injection (according to standard GLP toxicity study protocol for local injection) with vehicle control or VG161 at doses of 8.72 × 10^6^, 2.57 × 10^7^ or 2.57 × 10^8^ PFU/animal respectively (once daily, five days a week for 4 weeks, total 20 injections). For each group, the first 2 animals/sex/group were designated for the necropsy after the first round of dosing (Day 6). The middle 3 animal/sex/group were designated for the necropsy after 4 weeks of dosing (Day 27). The last 2 animals/sex/group were designated for necropsy after the 4-week recovery period following the last dosing (Day 56). Parameters evaluated in the study included mortality/morbidity, clinical signs, injection site reactions, body weights, body temperature, electrocardiogram, blood pressure, blood oxygen saturation, ophthalmoscopic examination, clinical pathology (hematology, coagulation, clinical chemistry, and urinalysis), T-lymphocyte, cytokines, C-reaction protein, complement, immunoglobulin, antibody, vector absorption, expression products in serum, tissue distribution of viral DNA, macroscopic and microscopic examinations. All animals were euthanized on Days 6, 27, or 56 and all received a complete necropsy examination.

### 2.16. Statistical Tests Employed in the Study

Data visualization and statistical analysis were performed using Microsoft Excel (Version 2007, Microsoft Corporation, Redmond, WA, USA) and GraphPad Prism (Version 8.4.3, GraphPad Software, San Diego, CA, USA), and all p values were determined using an unpaired *t*-test.

## 3. Results

### 3.1. Virus Characterization and In Vitro Validation of Oncolytic Activity

**Figure 2:**
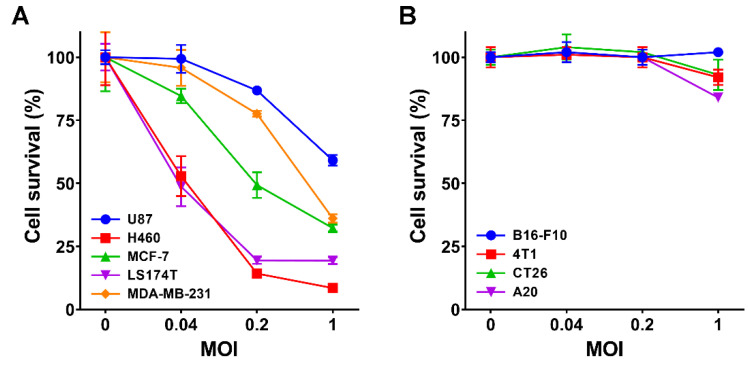
**Cytotoxicity of mutant viruses.** (**A**) The cytotoxic effect of VG161 virus was evaluated in a variety of human cancer cell monolayers including U87, H460, MCF-7, LS174T, and MDA-MB-231 at 72 h post infection and MOI of 0.04, 0.2, and 1. Cell survival percentage was quantified by MTT assay. (**B**) A panel of 4 different mouse tumor cell lines including B16-F10, 4T1, CT26, and A20 was infected with mVG161 virus at MOI 0, 0.04, 0.2, and 1. Cell viability was quantified using MTT assay at 72 h post infection. Error bars indicate SD.

The oncolytic capability of VG161 (Figure 1) was evaluated in five different human tumor cell lines and in four mouse tumor cell lines. Cells were infected with VG161 for 72 h at MOI ranging from 0.04 to 1, and cell viability was quantified by MTT assay. The VG161 virus displayed robust cell killing ability, with cell survival below 40% in all but one of the human-derived cell lines at MOI = 1 (Figure 2A). Cell killing was drastically curtailed in mouse tumor cells (Figure 2B), likely due to the reduced ability of mouse cells to support HSV-1 replication [24]. These results are consistent with cytotoxicity data for the parental virus HSV-345 (ICP34.5 deleted HSV-1 strain 17), which shows dramatically impaired cell killing in mouse tumor cell lines 4T1 and CT26 when compared to the human colon adenocarcinoma cell line LS174T (Figure S1A). The similar cytotoxicity profiles between VG161/mVG161 and the parental HSV-345 mutant demonstrate that the extensive genome engineering required to create VG161/mVG161 did not have a negative impact on viral growth characteristics. This is further corroborated by the observation of near-identical replication kinetics in VG161, mVG161, and the parental viruses VG160 and HSV-345 on two different tumor cell lines (Figure S1B,C).

### 3.2. In Vitro Transgene Expression and Characterization

**Figure 3:**
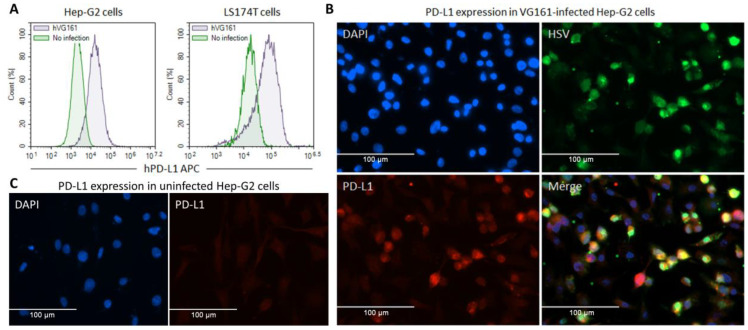
**Infection with VG161 leads to upregulation of PD-L1 expression.** (**A**) Hep-G2 and LS174T cells were seeded in a 12-well plate (2 × 10^5^ cells/well) and cultured at 5% CO2 and 37 °C overnight. The next day, half of the seeded cells were infected with hVG161 virus (MOI = 1) for 24 h. Cells were subsequently harvested and immunostained with purified rabbit monoclonal anti-human PD-L1 antibody plus APC-conjugated anti-rabbit IgG antibody and the expression level of PD-L1 was assessed by flow cytometry. (**B**,**C**) 5 × 10^4^/well of Hep-G2 cells were seeded in a 24 well plate with coverslip and incubated overnight at 37°C, followed by infection with hVG161 at MOI = 1 for 6 h. Cells were fixed in 4% PFA for 5 min and incubated in 3% skim milk in PBS-T for 30 min, followed by an overnight incubation at 4 °C with monoclonal mouse anti-HSV antibody (Abcam, 1:100 dilution) and with polyclonal rabbit anti-PDL1 antibody (Abcam, 1:100 dilution). The fixed cells were subsequently incubated with a mixture of fluorophore-labeled secondary antibodies (Alexa Fluor 488 goat anti-mouse and Alexa Fluor 568 goat anti-rabbit, Invitrogen, Burlington, ON, Canada; 1:500 dilution) in the dark for 1 h, counterstained with Hoechst (Sigma), and mounted on glass slides for imaging. (**B**) VG161-infected Hep-G2 cells. (**C**) Uninfected Hep-G2 cells.

Transgene expression was tested in H460 and LS174T human tumor cells by infecting the cells with VG161. Expression of the human IL-12/IL-15/IL-15RA cassette and human PD-L1 blocker (TF-Fc) was verified via ELISA by probing the cell lysates with antibodies against human IL-12, human IL-15, or human IgG, with strong expression evident in VG161-infected cells (Figure S2A). Payload expression by the mouse version of VG161 (mVG161) which encodes mouse IL-12 and the mouse version of PD-L1 blocker was verified by ELISA in the Vero cell line used for virus production due to poor virus growth in mouse tumor cell lines (Figure S3). To demonstrate the effectiveness of TF-Fc fusion protein as a PD-L1 blocker, we tested its ability to inhibit PD-1/PD-L1 interaction using ELISA (Figure S2B) and quantified IL-2 expression using a cell-based assay (Figure S2C). The resulting dose-dependent increase in IL-2 production by Jurkat cells co-cultured with PD-L1 expressing tumor cells in samples treated with TF-Fc peptide is consistent with successful blockage of PD-1/PD-L1 binding. Infection of Hep-G2 and LS174T cells with VG161 was also observed to strongly upregulate PD-L1 expression, which reinforces the need for concurrent expression of a checkpoint inhibitor (Figure 3).

### 3.3. Immune Activation by Virally Encoded Transgenes In Vitro

**Figure 4:**
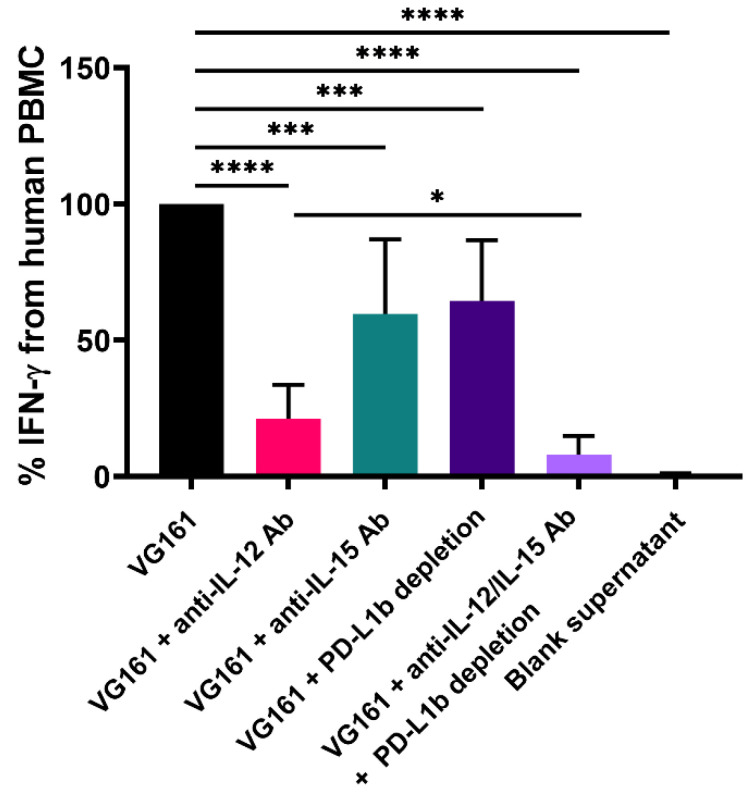
**IL-12, IL-15/IL-15RA, and PD-L1 blocker cooperatively enhance immune cell function in vitro.** PHA-activated human PBMCs were co-incubated with recombinant human PD-L1 protein and supernatant from VG161-infected Vero cells for 48 h. Antibody-mediated neutralization of IL-12 and/or IL-15 was carried out in conjunction with depletion of PD-L1 blocker. Co-incubation with supernatant from uninfected cells was used as negative control (blank supernatant). Human IFN-γ production was assessed by ELISA. * *p* < 0.05, *** *p* < 0.001, **** *p* < 0.0001. *p* values were computed using unpaired *t*-test. Error bars indicate SD.

Initial experiments demonstrated that healthy human donor PBMCs cocultured with supernatants from cells infected with VG161 or with the backbone virus VG160 that does not express IL-12, IL-15, IL-15RA, or the PD-L1 blocker peptide but contains all other modifications present in VG161 were activated by supernatants from VG161-infected cells in a concentration-dependent manner, while IFN-γ induced by VG160 remained near background levels (Figure S4). To further evaluate the combinatorial effect of virally encoded IL-12, IL-15/IL-15RA, and TF-Fc on immune cell function, we utilized targeted depletion of PD-L1 blocker, IL-12 and/or IL-15 expressed by VG161 (Figure 4). Activated human donor PBMCs were co-cultured with recombinant human PD-L1 protein and with supernatants from Vero cell monolayers infected with VG161, followed by ELISA to quantify production of human IFN-γ as described in Materials and Methods. Simultaneous use of both antibodies combined with PD-L1 blocker depletion drastically reduced IFN-γ production to near-background levels. Notably, individual application of either IL-12 or IL-15 neutralizing antibodies or selective depletion of PD-L1 blocker in PBMCs exposed to VG161-infected cell supernatants still yielded highly statistically significant (*p* < 0.001) reductions in IFN-γ levels ranging from approximately 35% after depleting the PD-L1 blocker to ~40% after administration of anti-IL-15 antibody and ~75% after treatment with the anti-IL-12 antibody. These data further demonstrate that IL-12 and IL-15 encoded by VG161 work together with the secretable TF-Fc peptide to stimulate immune cell activation and cytokine production.

### 3.4. VG161 Promotes Efficient Tumor Clearance In Vivo

**Figure 5:**
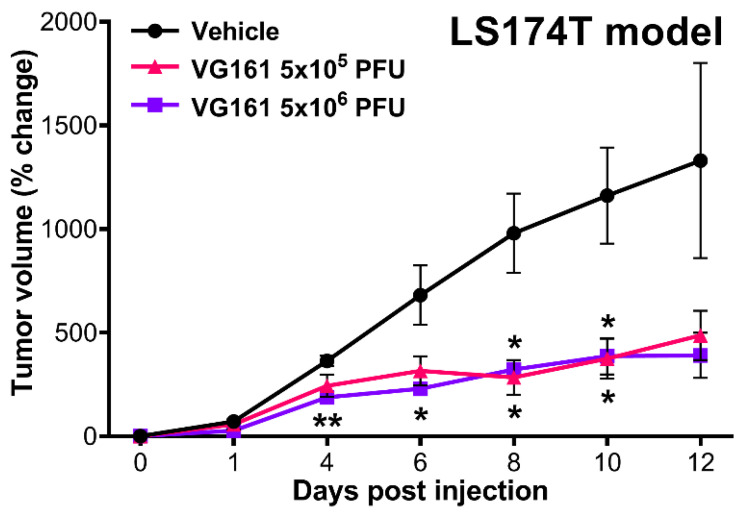
**In vivo efficacy of VG161 following intratumoral inoculation.** Three nude mice per group were subcutaneously implanted with 2 × 10^6^ LS174T human colon adenocarcinoma cells into the lower right flank, followed by a single intratumoral injection of either vehicle (PBS) control or 5 × 10^5^ or 5 × 10^6^ PFU/mouse of VG161. * *p* < 0.05, ** *p* <0.01. *p* values were computed using unpaired *t*-test.

In vivo efficacy of VG161 following intratumoral inoculation was further evaluated using an LS174T human colon adenocarcinoma model in nude mice (Figure 5). LS174T tumor cells were subcutaneously engrafted in mouse flanks and intratumorally injected once with either 5 × 10^5^ or 5 × 10^6^ PFU/mouse of VG161 or with vehicle control. Mice injected with both dosages of VG161 experienced a significant reduction in tumor growth when compared to vehicle-injected mice, with a statistically significant (*p* < 0.05) difference observed in the low dose VG161-injected group at 8 and 10 days post-injection and in the high dose VG161-injected group at 4, 6, 8, and 10 days post-injection.

**Figure 6:**
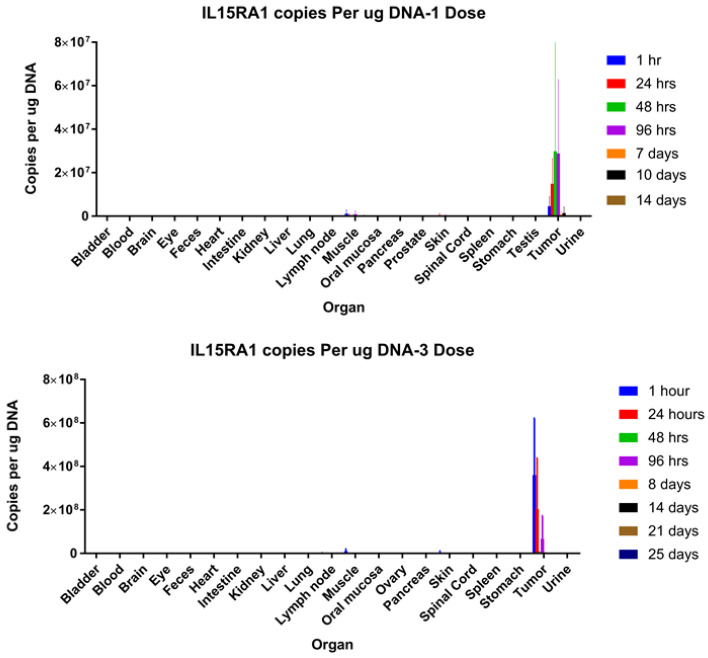
**Virus biodistribution.** Nude mice bearing LS174T tumors were injected intratumorally with either 1 or 3 doses of VG161 (1 dose = 5 × 10^7^ PFU/mouse). Mice were euthanized at different time points, and genomic DNA was isolated from these organs and subjected to qPCR to quantify the viral copy number using the codon optimized IL-15RA1 gene due to its specificity to VG161.

Virus biodistribution at different time points post-injection in an LS174T human xenograft tumor model was also measured by qPCR, revealing that virus from a single injection persists at high levels for up to 96 h post-injection and remains localized to the tumor mass even if multiple injections are administered (Figure 6). These data demonstrate that, in the absence of a functional immune system, VG161 can exert therapeutic efficacy solely from its potent and selective oncolytic activity.

### 3.5. mVG161 Induces a Potent Anti-Tumor Immune Response In Vivo

**Figure 7:**
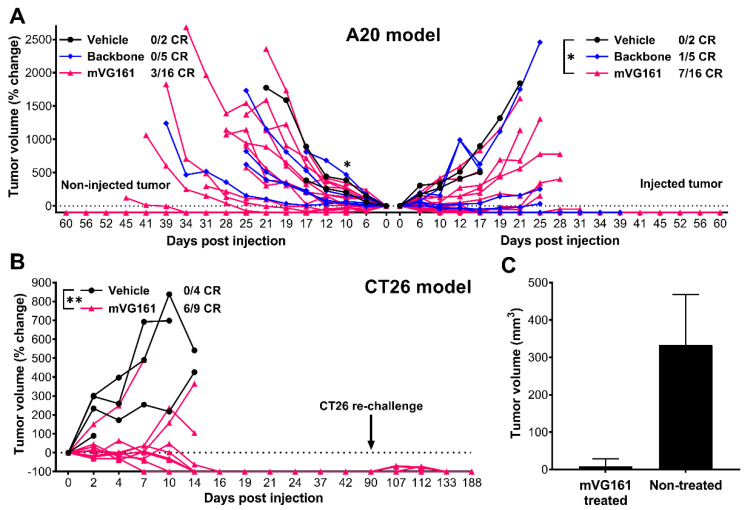
**In vivo efficacy of a murine version of VG161 (mVG161) following intratumoral inoculation.** (**A**) A20 cells were subcutaneously implanted into immunocompetent BALB/C mice in both sides of lower flanks (5 × 10^6^ A20 cells per flank). 5 × 10^6^ PFU/mouse/day of either mVG161 or VG160 backbone virus (version of VG161 without payload) was injected once per day for 5 consecutive days into tumors on one side only. 16 mice were treated with mVG161, 5 mice were treated with VG160, and 2 mice were treated with vehicle (PBS) control. (**B**) Thirteen immunocompetent BALB/C mice were subcutaneously implanted with 1 × 10^6^ CT26 cells/mouse into the lower flanks, with 9 animals randomly assigned to the mVG161 treatment group (5 × 10^6^ PFU/mouse injected 5 times) and another 4 animals to the vehicle (PBS) control group. At 90 days post injection, the surviving 6 mice in the mVG161-treated group were re-implanted with 1 × 10^6^ CT26 cells in the same location. (**C**) Tumor sizes 7 days after CT26 re-challenge in mVG161-treated animals compared to age-matched (28 weeks old) control mice that were not treated with mVG161. CR = complete response. * *p* < 0.05, ** *p* < 0.01. *p* values were computed using unpaired *t*-test. Error bars indicate SD.

To investigate the role of anti-tumor immune response elicited by VG161, a dual tumor A20 model was used (Figure 7A). Immunocompetent BALB/c mice bearing subcutaneous A20 murine B cell lymphoma/sarcoma tumors in both flanks were injected intratumorally on the left side with either PBS control, backbone virus (VG160), or mVG161 (containing murine versions of IL-12 and PD-L1 blocker) while the tumors on the right side were not treated. In animals treated with mVG161, statistically significant (*p* < 0.05) tumor regression was observed on the injection side at each measured timepoint, with 7 out of 16 animals showing a complete response and another animal with a partial response (tumor volume was reduced by 50.9% compared to baseline). This was followed by a delayed but notable regression on the non-injection side, with 3 out of 16 mVG161-treated animals achieving complete tumor clearance and reaching statistical significance (*p* < 0.05) by 10 days post injection when compared to PBS-treated controls. Interestingly, in animals that received the backbone oncolytic virus lacking immune stimulating factors (VG160), the tumor inhibitory effect was greatly reduced, especially on the untreated side. Immune-mediated abscopal clearance of non-injected distal tumors strongly suggests that the anti-tumor effect displayed by the mVG161 virus when compared to VG160 is mediated by cytokine and/or PD-L1 blocker expression by mVG161 instead of by direct oncolysis.

To determine if mVG161 can elicit long-lasting anti-tumor immunity, a tumor re-challenge test was performed in a syngeneic CT26 model (Figure 7B). BALB/c mice were implanted subcutaneously with CT26 murine colon carcinoma cells and injected intratumorally with either mVG161 or vehicle control. Complete tumor regression was observed by 16 days post injection in 6 out of 9 animals treated with mVG161, while 3 of the 4 PBS-treated animals were euthanized due to tumor burden before 16 days post injection. The fourth PBS-treated animal and the 3 dead mVG161-treated animals were euthanized early because the tumors showed signs of ulceration, in accordance with our animal care protocols. The difference in response between PBS-treated and mVG161-treated animals was highly statistically significant (*p* < 0.01) at each measured timepoint prior to loss of the control group. The surviving six mice were re-challenged 90 days later by implanting additional CT26 tumor cells at the same site, but tumors failed to develop, and all of the re-challenged mice survived for the entire 188-day duration of the experiment. At the same time, CT26 cells successfully established tumors in age-matched naïve mice up to 29 weeks old, indicating that older mice can still support CT26 tumor growth in the absence of treatment with mVG161 (Figure 7C).

**Figure 8:**
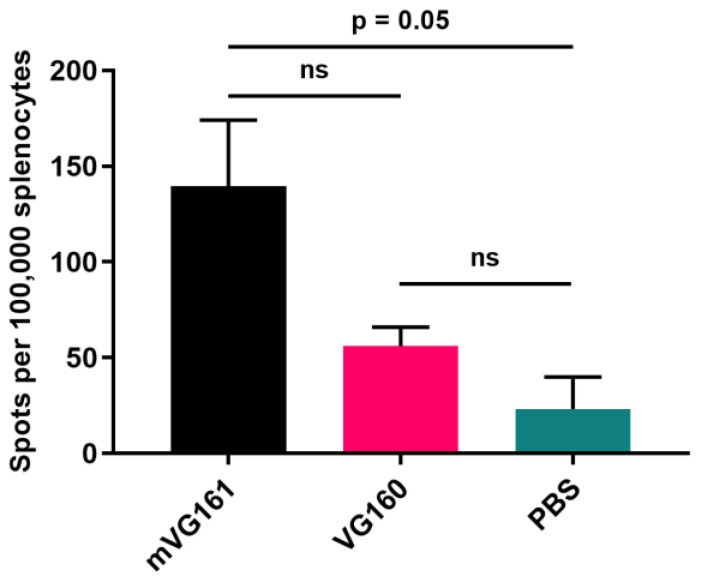
**ELISpot assay to evaluate T cell activity in spleens from tumor bearing mice treated with mVG161.** BALB/c mice were subcutaneously implanted with 1 × 10^6^ CT26 tumor cells into the lower right flank, followed by multiple injections of 5 × 10^6^ PFU/mouse/day, for 5 consecutive days, of either mVG161, VG160, or PBS control. Mouse IFN-γ ELISpot assay was performed on splenocytes collected from CT26 tumor bearing mice at 5, 7, and 9 days post-injection and exposed to CT26 cells (results shown are from 5 days post treatment). Quantitative results are graphed with 2 mice per group. *p* values were computed using unpaired *t*-test. Error bars indicate SD.

The anti-tumor immunity induced by mVG161 was further demonstrated by ELISpot assay performed on splenocytes collected from CT26 tumor bearing mice between 5- and 9-days post-injection and stimulated with CT26 cells overnight as described in the Materials and Methods (Figure 8). The results indicate that mVG161 markedly increased the numbers of IFN-γ secreting cells compared to those elicited by the backbone virus, although the magnitude of this effect did not quite rise to the level of statistical significance. However, the results do suggest that, unlike VG160, the combination of immunomodulatory payloads in mVG161 is better able to stimulate the clonal expansion of anti-tumor T cells and/or facilitate T cell tumor-epitope spread when compared to PBS-treated control.

### 3.6. mVG161 Alters the Tumor Microenvironment (TME)

**Figure 9:**
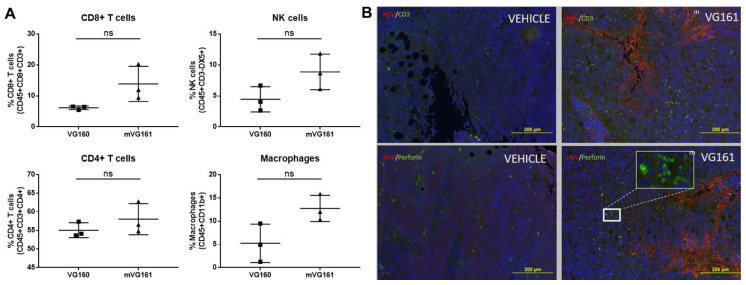
**Effect of mVG161 treatment on intratumoral lymphocyte populations.** BALB/c mice were subcutaneously implanted with 1 × 10^6^ CT26 tumor cells, followed 8 days later by 5 consecutive injections of PBS (vehicle), VG160 backbone, or mVG161 virus (5 × 10^6^ PFU/mouse/day). Tumors were harvested 24 h after final injection. (**A**) Percentages of different subsets of T cells, NK cells, and macrophages within the tumor mass were analyzed by flow cytometry by gating on CD45+ leukocytes and then looking at the population of CD8+/CD4+ T cells, NK cells, and macrophages based on different surface markers (ns = not significant). (**B**) Immunohistochemical analysis was performed on sections of excised CT26 tumor treated with either PBS control (vehicle) or with mVG161 using monoclonal antibodies against CD3 and perforin and polyclonal antibodies against HSV-1. *p* values were computed using unpaired *t*-test.

To elucidate the precise nature of this anti-tumor immune response, we utilized flow cytometry to analyze cells isolated from excised CT26 tumors taken from BALB/c mice that were treated with either VG160 backbone virus or mVG161 virus. mVG161 injection was correlated with increases in multiple populations of immune cells when compared to VG160 virus, including macrophages, NK cells, and both CD4+ and CD8+ T cells (Figure 9A).

**Figure 10:**
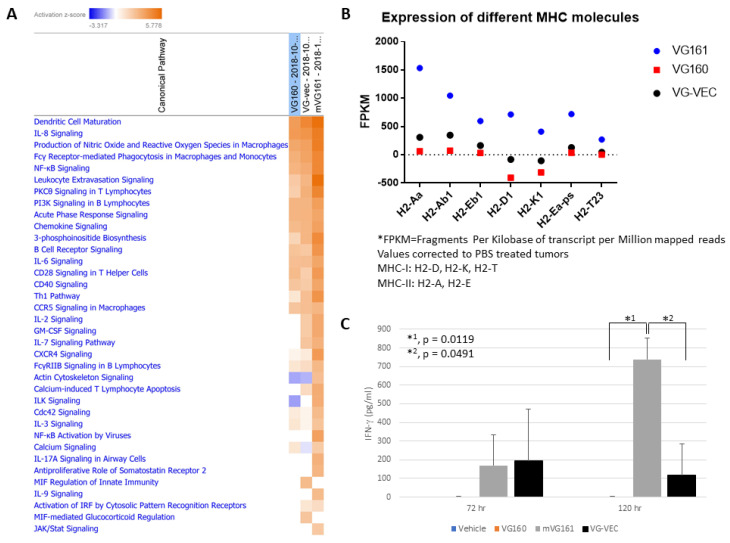
**VG161 multi-factor payload elicits a stronger immune response than GM-CSF.** (**A**) BALB/c mice were subcutaneously implanted with 1 × 10^6^ CT26 tumor cells and subsequently treated with 5 daily injections (5 × 10^6^ PFU/mouse/day) of VG161, VG160 (backbone) or VG-VEC (VG160 expressing GM-CSF). Tumors were harvested 24 h after the final virus injection, RNA was isolated and purified, followed by transcriptome sequencing using the Illumina NGS platform. Data were analyzed using Qiagen Ingenuity Pathway Analysis (IPA) software to evaluate activation of immunostimulatory pathways in each treatment group. (**B**) Expression of MHC molecules in each treatment group was also quantified, and the over-expression of some MHC targets was validated by RT-qPCR. (**C**) BALB/c mice were implanted with 1 × 10^6^ CT26 tumor cells and injected 5 times daily with the indicated viruses (5 × 10^6^ PFU/mouse/day). Mouse serum was collected either 72 h or 120 h after the final injection, and mouse IFN-γ production was assessed by ELISA assay. *p* values were computed using an unpaired *t*-test. Error bars indicate SD.

Infected tumor sections were also stained and immunohistochemically imaged, displaying enrichment with CD3 and perforin compared to vehicle-treated controls, suggesting the presence of tumor-infiltrating lymphocytes within the TME (Figure 9B). Differential gene expression in implanted CT26 tumors treated with mVG161 compared to equivalent tumors injected with VG160 (Figure S5) revealed that infection with mVG161 bearing IL-12, IL-15, and IL-15RA transgenes powerfully stimulates a prototypical Th1 immune response (e.g., IFN-γ, TNF, and IL18). Moreover, the signature of anti-tumor immunity elicited by VG161 is more robust than both VG160 and VG-VEC (VG160 backbone expressing GM-CSF) based on transcriptome sequencing data and differences in IFN-γ production and MHC molecule expression in CT26 tumor-bearing mice treated with each respective OV (Figure 10). Taken together, these data suggest that VG161 profoundly modulates the TME to drive a robust anti-tumor immune response.

### 3.7. VG161 Displays a Robust Safety Profile in Primates

Both acute (single dose) and repeated injection toxicity studies were performed using cynomolgus monkeys (*Macaca fascicularis*) in a GLP-compliant laboratory. The single dose toxicity study comprised a total of eight monkeys (4 animals/sex) assigned to four groups (1 animal/sex/group) that were treated via single intramuscular injection with either vehicle control or VG161 at doses of 2.97 × 10^7^, 2.97 × 10^8^, and 2.97 × 10^9^ PFU/animal, respectively, followed by a 14-day observation period. All animals were euthanized on Day 15 and received a complete necropsy examination. Neither mortality nor morbidity was noted in any animals throughout the study. No treatment-related abnormal findings/changes in clinical observations, cage-side observations, body weights (Figure S6), body temperature, electrocardiogram parameters, clinical pathology, T-lymphocytes, or cytokines were in evidence. No test article related macroscopic examination abnormalities were noted in any of the treated animals. The maximum tolerated dose (MTD) for cynomolgus monkeys was determined to be equal to or greater than 2.97 × 10^9^ PFU/animal.

The repeat dose toxicity study comprised seven animals/sex/group that were treated via repeated intramuscular injection with either vehicle control or VG161 at doses of 8.72 × 10^6^, 2.57 × 10^7^, or 2.57 × 10^8^ PFU/animal, respectively. In each group, two animals were euthanized and necropsied after the first set of injections on day 6, an additional three animals were euthanized and necropsied after 4 weeks of injections on day 27, and the final two animals were euthanized and necropsied on day 56 after a 4-week recovery period following the final injection. For the 4-week toxicity study of VG161 followed by a 4-week recovery period, neither mortality nor morbidity was noted in any animals throughout the study. No significant changes in body weight were observed even before the start of the 4-week recovery period (Figure S6). No abnormal changes were noted in main organs such as heart, liver, lung, kidney, and spinal cord of each group. The no observed adverse effect level (NOAEL) was determined to be 2.57 × 10^8^ PFU/animal.

## 4. Discussion

Broadly effective cancer immunotherapy remains elusive, hampered by tumor genetic diversity, a dearth of neoantigens, and an immunosuppressive tumor microenvironment. Immunologically “cold” tumors are characterized by localized depletion of cytotoxic effector T cells and NK cells, coupled with elevated levels of myeloid-derived suppressor cells (MDSCs) and regulatory T cells, often exacerbated by reduced pH in the tumor microenvironment (reviewed in [25]).

Besides directly killing tumor cells, local administration of OVs generates localized inflammation in the tumor microenvironment capable of attracting multiple immune effector cells to convert immunologically “cold” tumors into a “hot” immunogenic state. HSV-1 is a highly immunogenic OV, facilitating the recruitment of M1 monocytes/macrophages [26] and antagonizing Treg-mediated immunosuppression [27]. However, the immunity elicited by locally injected OVs such as HSV-1 is mainly anti-viral, and thus often insufficient to generate a lasting anti-tumor immunity [28]. Attempts have been made at systemic delivery of HSV, most notably using NV1020, which is an attenuated HSV-1 derived from strain F and characterized by a deletion of both UL56 and the promoter of UL24, as well as the deletion of one copy of ICP0, ICP4, and ICP34.5. The expression of viral thymidine kinase is further altered in NV1020 to be regulated by the ICP4 promoter [29]. While systemic delivery of oncolytic virus is an important area for future development, the efficacy of intravenously delivered HSV has not been very promising in clinical trials, largely due to rapid antibody-mediated neutralization of the virus. On the other hand, there exists pre-clinical evidence [30], as well as data from multiple trials utilizing Talimogene laherparepvec [31], that have shown an abscopal effect by intratumorally injected OVs, thus suggesting that locally delivered OVs can also generate a systemic therapeutic response.

To enhance the OV’s ability to mount an effective and lasting immune response against tumors, we have developed and tested the VG161 replication-competent oncolytic virus platform expressing a suite of immunomodulators tailored to cooperatively promote a systemic anti-tumor immune response. Vector safety was enhanced by deletion of the RL1 gene encoding the neurovirulence factor ICP34.5, which antagonizes the interferon response-induced shutdown of protein synthesis in infected cells by stimulating the dephosphorylation of the alpha subunit of eukaryotic translation initiation factor 2 (eIF-2). Since the interferon response pathway is typically defective in tumor cells, the deletion of ICP34.5 also confers a measure of tumor selectivity [32,33].

We incorporated genes encoding secretable immune modulators IL-12, IL-15, IL-15RA, and a PD-L1 blocking peptide into VG161 to promote durable anti-tumor immunity by enhancing T_H_1 polarization and the inflammatory response within the tumor microenvironment while avoiding potential toxicity associated with systemic administration of cytokines [[34], reviewed in [35]]. Inclusion of granulocyte-macrophage colony-stimulating factor (GM-CSF) has been a popular strategy to enhance immune activation in the context of an oncolytic virus, but our results (Figure 10) showed that GM-CSF alone may not be sufficient to generate an inflammatory immune response. In addition, GM-CSF may actually be counterproductive because GM-CSF has been implicated as a major factor promoting stimulation of MDSCs [36,37,38,39,40]. IL-12 is a cytokine shown to possess dramatic antitumor effects in preclinical studies through enhanced CTL activity and elevated production of cytokines such as IFN-γ triggered by differentiation of T helper 1 cells [11,14]. While IL-2 is considered a prototype for γ-chain cytokines and is the quintessential growth factor for T cells, we have opted to use IL-15 instead to combine with IL-12 as a better candidate for cancer immunotherapy for several reasons. Among these, IL-15 protects T cells from IL-2 induced Activation Induced Cell Death (AICD), and while IL-2 is important in maintaining regulatory T cells that suppress CD8+ T cell responses, IL-15 does not have this effect [16,41]. Moreover, numerous studies suggest that IL-15 is ideally suited for combination therapy [17], and we have reported that this cytokine synergizes with components of the HSV-1 molecular structure to activate NK cells [18,19]. Pre-activation of NK cells with IL-18, IL-12, and IL-15 prior to adoptive NK cell transfer leads to high levels of IFN-γ production coupled with enhanced NK cell survival [42], while co-administration of IL-15 has been shown to amplify the anti-tumor activity of IL-12 in a murine metastatic melanoma model [16,43]. IL-15RA was included because it acts as a soluble IL-15 agonist, forming stable complexes that prolong the in vivo half-life of IL-15 and enhance its biological activity [17,22,44].

Tumors take advantage of immune checkpoint pathways to shut down T cell activity and inhibit the anti-tumor immune response, mediated by proteins such as PD-L1 and CTLA-4. Immune checkpoint blockade is a promising therapeutic strategy to restore immunosurveillance and multiple checkpoint inhibitors have been successfully used for cancer immunotherapy [45,46,47,48,49,50]. Several reports have indicated that viral oncolysis strongly induces PD-L1 expression in primary and metastatic tumors [51,52], and we observed a similar pattern of PD-L1 upregulation after infecting Hep-G2 and LS174T cells with VG161 (Figure 3). Blocking the PD1/PD-L1 pathway was also an ideal target for VG161 due to higher response rates and lower severity of adverse events compared to CTLA-4 blockade [53,54]. Additionally, combinatorial treatment with IL-12 and checkpoint inhibitors was shown to eliminate advanced glioblastoma tumors in a T cell dependent manner [55]. Antibodies against PD-1 have already been used successfully in conjunction with oncolytic HSV-1 injection [9,56], thus making direct expression of a PD-1 antagonist a logical next step in OV development.

While oncolytic viruses carrying individual IL-12 [57,58,59], IL-15 [59,60], or a checkpoint inhibitor [61] as payloads are not a novel concept, our work represents the first time that all three payloads have been delivered simultaneously. Although it would be difficult to directly compare our findings with those in the literature, our results support the presence of a potent immunostimulatory effect when combining IL-12, IL-15/IL-15RA, and the PD-L1 blocker in the context of an oncolytic virus, as evidenced by significantly higher levels of immune activation by VG161 in vitro when compared to VG161 that has been subjected to selective depletion of its payloads using neutralizing antibodies.

To distinguish between two mechanisms of tumor destruction by VG161 (direct oncolysis vs. immune-mediated tumor clearance), we used both athymic nude mice and immunocompetent BALB/c mice for in vivo testing. Tumor clearance in nude mice is largely dependent on virus replication and direct oncolysis, which can be inhibited due to intracellular anti-viral mechanisms. Infected cells and free virus particles are much more likely to be recognized and cleared by a functional immune system, thus the immunomodulatory payload delivered by VG161 can play a much larger role in immunocompetent mice where lytic replication of HSV is limited.

VG161 efficiently promoted tumor clearance in both syngeneic mouse models despite exhibiting poor in vitro cytotoxicity on the CT26 and A20 murine tumor cell lines. Moreover, treated CT26-implanted mice were able to survive tumor re-challenge, and mice implanted with bi-lateral A20 tumors consistently cleared both tumors following unilateral injection with VG161 while succumbing to tumor burden after treatment with a variant of VG161 lacking IL-12, IL-15/IL-15RA, and the PD-L1 blocker (VG160). Examination of tumors treated with VG161 compared to equivalent tumors exposed to VG160 provides compelling evidence that VG161 induced a potent systemic anti-tumor immune response in vivo, revealing increased infiltration of NK cells, macrophages, and both CD4+ and CD8+ tumor-specific T cells. Higher numbers of tumor specific splenic T cells were also demonstrated by ELISpot assay in CT26 tumor bearing mice treated with VG161 compared to VG160 or vehicle treated mice. Finally, and as a requirement for filing an IND application, GLP toxicity studies were performed to evaluate single-dose and repeat-dose toxicity of VG161 after administration to cynomolgus monkeys by intramuscular injection. In both cases, no treatment-related abnormal clinical observations were noted in any animals, and no anomalies were observed in a battery of laboratory and clinical tests.

In conclusion, we have demonstrated that a rational combination of payloads delivered by VG161 can induce pro-inflammatory changes in the tumor microenvironment, antagonize immune checkpoints, and render tumors more susceptible to immune destruction.

## Supplementary Materials

Supplementary materials can be found at https://www.mdpi.com/2227-9059/8/11/484/s1. Figure S1: Cytotoxicity and replication kinetics of mutant viruses. Figure S2: In vitro characterization of IL-12, IL-15, and PD-L1 blocker expressed by VG161. Figure S3: In vitro characterization of IL-12, IL-15, and PD-L1 blocker expressed by mVG161. Figure S4: Concentration dependent effect of supernatants from VG161-infected cells on PBMCs. Figure S5: qPCR array. Figure S6: Primate body weight remains stable during toxicity studies.
