# Supplementary material for: Induction of Durable Antitumor Response by a Novel Oncolytic Herpesvirus Expressing Multiple Immunomodulatory Transgenes
Source: Biomedicines. 2020 Nov 9;8(11):484. doi: 10.3390/biomedicines8110484 (PMC7695276; doi:10.3390/biomedicines8110484)
Supplement: Supplementary file 1 [file biomedicines-08-00484-s001.zip › biomedicines-966993-supplementary.docx]

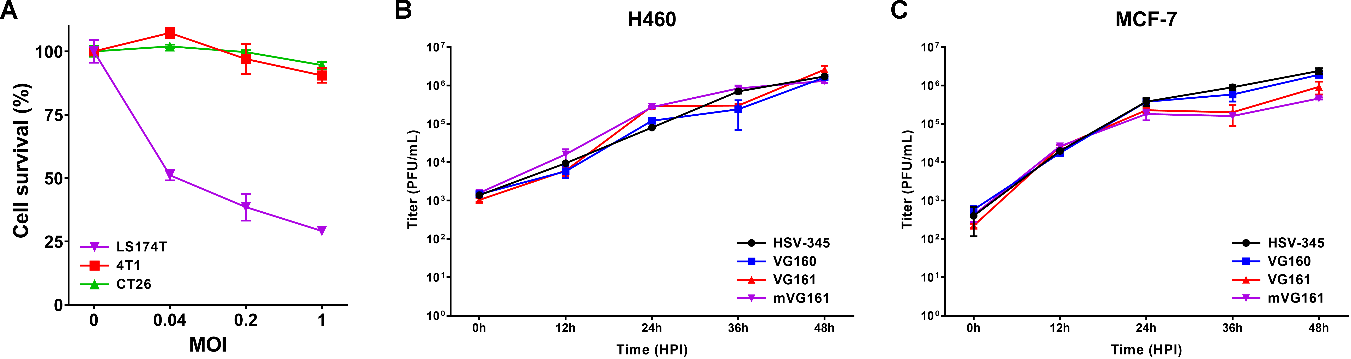


**Supplementary Figure S1.** **Cytotoxicity and replication kinetics of mutant viruses.** (A) The cytotoxic effect of HSV-345 virus was evaluated in human tumor cell line LS174T and mouse tumor cell lines 4T1 and CT26 at 72 hours post infection and MOI of 0.04, 0.2, and 1. Cell survival percentage was quantified by MTT assay. (B, C) H460 and MCF-7 human cancer cell monolayers were infected with either VG161, mVG161, or the parental backbone viruses VG160 and HSV-345 at MOI=0.1, and samples were harvested at the indicated timepoints. Viral titers at each timepoint were determined by plaque assay. Error bars indicate SD.


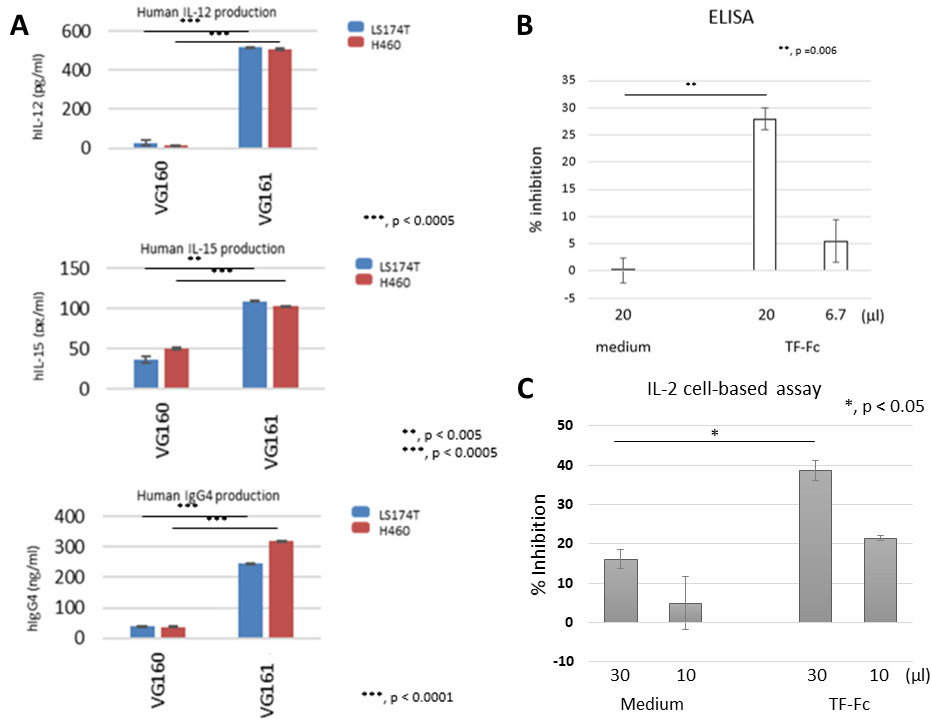


**Supplementary Figure S2. *In vitro* characterization of IL-12, IL-15, and PD-L1 blocker expressed by VG161.** (A) H460 human lung cancer cells and LS174T colon cancer cells were infected with VG161 or its backbone virus VG160 (MOI=1) for 24 hours. Transgene expression was quantified by ELISA. (B) Harvested TF-Fc peptide-containing supernatants from transfected 293FT cells were mixed with recombinant human PD-1 Fc and bound to a human PD-L1 Fc coated 96-well Immuno Maxisorp flat bottom plate. Binding was detected via a biotinylated anti-PD-1 antibody, streptavidin-horseradish peroxidase (HRP), and 3,3’,5,5’-Tetramethylbenzidine (TMB) substrate. Absorbance measurements were collected at 450 nm via a plate reader. The increased percent (%) of human PD-1/PD-L1 inhibition was compared to no peptide sample. (C) Cell-based assay results of TF-Fc peptide treatment. 5 × 10^4^ Jurkat T cells were activated with 1 µ/ml of PHA and 50 ng/ml of PMA and co-cultured with 1 × 10^5^ PD-L1-expressing tumor cells mixed with PD-L1 blocking peptide-containing supernatants at 37°C for 48 hours. After 48 hours, cell culture supernatants were harvested and IL-2 production from Jurkat T cells was assessed by IL-2 ELISA. P values were computed using unpaired t-test. Error bars indicate SD.


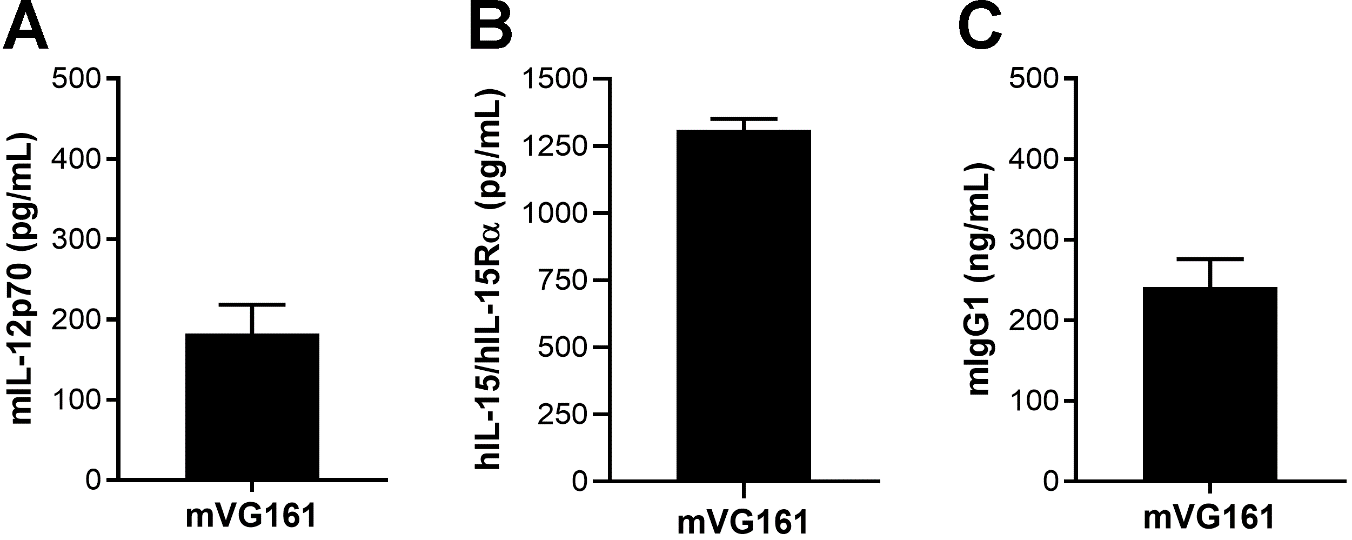


**Supplementary Figure S3. *In vitro* characterization of IL-12, IL-15, and PD-L1 blocker expressed by mVG161.** (A) Vero cells were infected with mVG161 (MOI=1) for 48 hours. Transgene expression was quantified by ELISA. Error bars indicate SD.


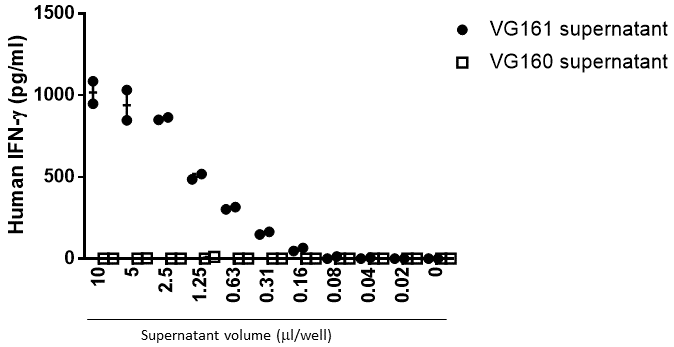


**Supplementary Figure S4. Concentration dependent effect of supernatants from VG161-infected cells on PBMCs.** 2 × 10^4^ cells/well human PBMCs were cultured with PHA for 48 hours and subsequently co-incubated with different volume of VG161 or VG160 supernatant in 96-well plate for 48 hours. Human IFN-γ production was assessed by ELISA assay.


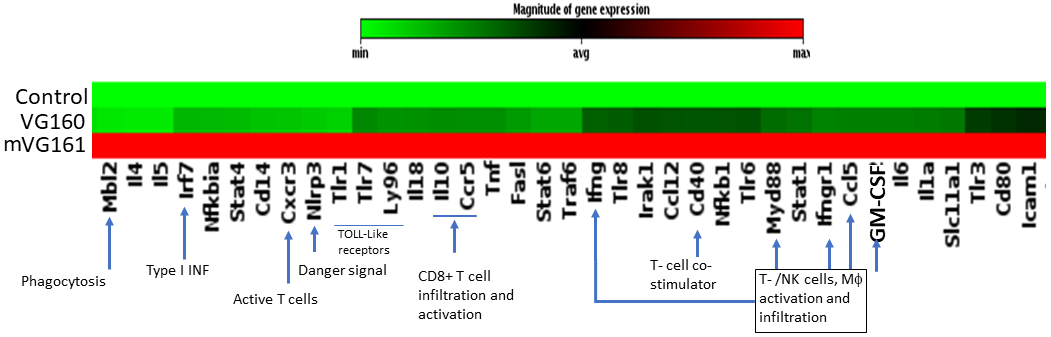


**Supplementary Figure S5. qPCR array.** BALB/c mice were subcutaneously implanted with 1 × 10^6^ CT26 tumor cells and subsequently treated with 5 daily injections of mVG161, the control virus VG160, or PBS (1 × 10^7^ PFU/mouse/day). The tumors were harvested 24 hours after the last virus injection, RNA was isolated and purified, followed by gene expression profiling using the Mouse Innate & Adaptive Immune Responses RT^2^ Profiler™ PCR Array from Qiagen. Differential expression of innate and adaptive immunity related genes in tumors treated with mVG161 compared to those treated with VG160 and PBS is represented as a heat map. The overexpression of indicated targets was validated by RT-qPCR.


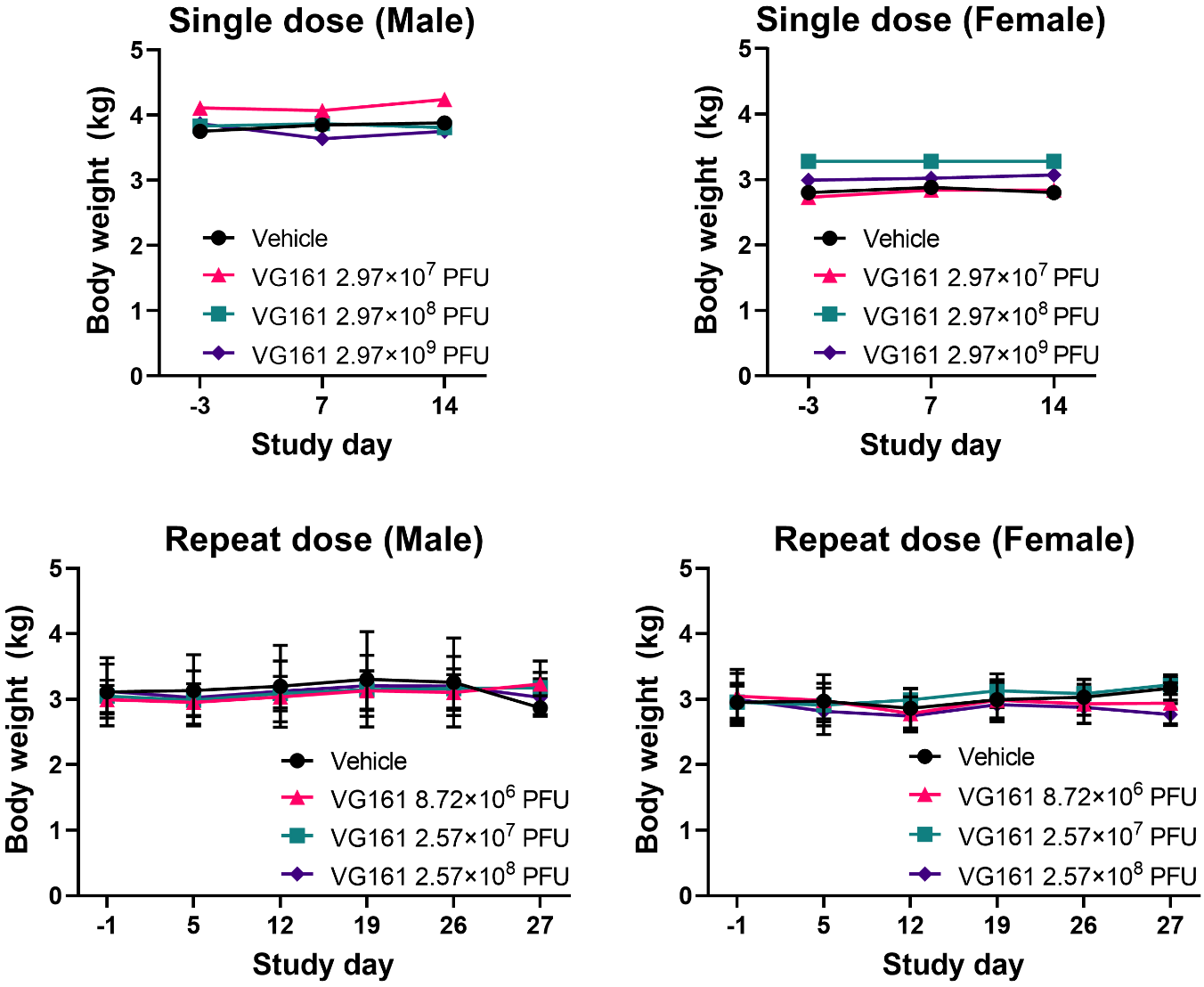


**Supplementary Figure S6. Primate body weight remains stable during toxicity studies.** In the single dose study, 8 cynomolgus monkeys (4 animals/sex) were assigned to 4 groups (1 animals/sex/group) and treated by administering a single intramuscular injection with either vehicle control or VG161 at the indicated doses. In the repeat dose study, 7 animals/sex/group were treated via repeated intramuscular injections with either vehicle control or VG161 at the indicated doses. Negative study day values indicate that the body weight measurement was taken 3 days before injections were administered in the single dose study and 1 day before injections were administered in the repeat dose study. Error bars indicate SD.
